# Evaluating an integrated primary care approach to improve well-being among frail community-living older people: A theory-guided study protocol

**DOI:** 10.1186/s12877-018-0832-1

**Published:** 2018-08-03

**Authors:** Lotte Vestjens, Jane M. Cramm, Erwin Birnie, Anna P. Nieboer

**Affiliations:** Erasmus School of Health Policy & Management, Erasmus University Rotterdam, P.O. Box 1738, 3000 DR Rotterdam, the Netherlands

**Keywords:** integrated care, primary care, frailty, elderly, theoretical model, quasi-experimental design, (cost)effectiveness, quality of care, well-being

## Abstract

**Background:**

A major challenge in primary healthcare is the substantial increase in the proportion of frail community-dwelling older persons with long-term conditions and multiple complex needs. Consequently, a fundamental transformation of current models of primary care by means of implementing proactive integrated care is necessary. Therefore, an understanding of the effects of integrated primary care approaches and underlying mechanisms is essential. This article presents the design of a theory-based evaluation of an integrated primary care approach to improve well-being among frail community-living older adults, which is called “*Finding and Follow-up of Frail older persons” (FFF)*.

First, we present a theoretical model to facilitate a sound theory-guided evaluation of integrated primary care approaches for frail community-dwelling older people. The model incorporates interrelated elements of integrated primary care approaches (e.g. proactive case finding and self-management support). Efforts to improve primary care should integrate these promising components to assure productive patient-professional interactions and to improve well-being. Moreover, cognitive and behavioral components of healthcare professionals and patients are assumed to be important. Second, we present the design of the study to evaluate the FFF approach which consists of the following key components: (1) proactive case finding, (2) case management, (3) medication review, (4) self-management support, and (5) working in multidisciplinary care teams.

**Methods:**

The longitudinal evaluation study has a matched quasi-experimental design with one pretest and one posttest (12 month follow-up) and is conducted in the Netherlands between 2014 and 2017. Both quantitative and qualitative methods are used to evaluate effectiveness, processes, and cost-effectiveness. In total, 250 frail older persons (75 years and older) of 11 GP (general practitioner) practices that implemented the FFF approach are compared with 250 frail older patients of 4 GP practices providing care as usual. In addition, data are collected from healthcare professionals. Outcome measures are based on our theoretical model.

**Discussion:**

The proposed evaluation study will reveal insight into the (cost)effectiveness and underlying mechanisms of the proactive integrated primary care approach FFF. A major strength of the study is the comprehensive evaluation based on a theoretical framework. The quasi-experimental design presents some challenges.

## Background

Population aging is challenging the delivery of primary care for older people. In the Netherlands, the number of people aged 65 years and older will increase from 3 million in 2015 (17.8% of the total population) to 4.7 million in 2060 (26% of the total population) [1]. The condition of frailty is considered an increasingly problematic consequence of population aging [2]. The main feature of frailty is the increased vulnerability to stressors resulting from impairments in several systems leading to decreased reserve capacity [3–5]. The level of frailty can be placed on a continuum ranging from not frail to frail [6]. In addition, frailty appears to be a dynamic state in which people can become less or more frail over time [7]. Frail people have an increased risk of negative (health) outcomes, like institutionalization, disability, mortality, and the development or progression of (multiple) chronic conditions [4, 8–12]. Older people can simultaneously have multiple chronic conditions, be frail and disabled, which increases the complexity of their healthcare needs [12]. Internationally, one important challenge to healthcare is the substantial increase in the proportion of frail older people with often multiple complex needs [13, 14] and an increased healthcare utilization [15]. Despite the substantial increase of frail older people with multiple complex needs, living independently in the community and avoiding or delaying institutional care is the avowed ambition of policy makers [15]. This has led to a decline in the proportion of older people in homes for the elderly and nursing homes [16]. Furthermore, most older people these days prefer to remain living at home for as long as possible [17, 18]. The government increasingly expects frail older people to arrange their own care, e.g. informal care, and limits access to long-term care facilities. Consequently, care for older people is increasingly being delivered in the primary care setting by GP (general practitioner) practices [15]. In the Netherlands, the GP has a central and exceptional role in healthcare, since GPs function as primary care gatekeepers for secondary healthcare [19]. The current primary care system is fragmented and reactive, and neither able to cope effectively with the increasing demands for healthcare, nor to improve well-being of frail community-dwelling older people [19–21].

As a consequence, to meet the needs of frail older people and improve their well-being, primary healthcare systems are changing [15] and many innovative integrated primary care approaches have emerged to provide optimal care [22]. In essence, stable well-being is when frail older people have the psychological, social and physical resources they need to meet a particular psychological, social and/or physical challenge [23]. Health care systems need to be supportive of such challenges. Studies evaluating innovative primary care approaches, however, show inconsistent results with respect to effectiveness. Moreover, assessment of cost-effectiveness of primary care approaches is often ignored [24–30]. Furthermore, a sound understanding of the effects of integrated primary care approaches and underlying mechanisms explaining effectiveness is lacking. This calls for a theory-based evaluation of such approaches.

The present study focuses on (1) the development of a theoretical model to facilitate the evaluation of integrated primary care approaches for frail older patients and to understand the underlying mechanisms explaining (lack of) effectiveness, and (2) the development of a theory-guided study protocol to evaluate a proactive integrated primary care approach to improve well-being of frail community-dwelling older people.

### A theoretical model to facilitate the evaluation of integrated primary care approaches

**Fig. 1:**
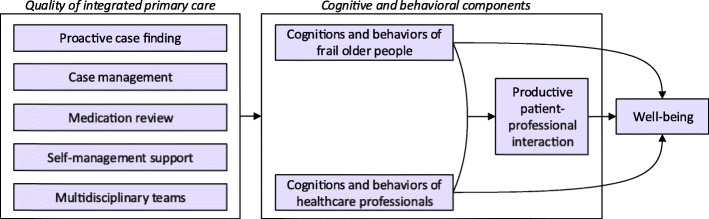
Theoretical model to facilitate a theory-based evaluation of integrated primary care approaches for frail community-dwelling older people

Many interventions to improve healthcare entail complex changes in daily routines and organization of healthcare, and collaboration among healthcare professionals of different disciplines. Moreover, changes in the behaviors of patients are necessary. It is important to incorporate theoretical assumptions in the development and evaluation of innovative approaches to improve patient care because it provides insight into the underlying mechanisms of integrated primary care approaches and insight into the complexity of changing healthcare practices [31]. Therefore, a theory-guided evaluation of an innovative integrated primary care approach is proposed (see Fig. 1). In Fig. 1 we show how proposed interrelated components of care delivery are presumed to influence cognitions and behaviors of frail older patients and healthcare professionals. These cognitions and behaviors are assumed to foster productive patient-professional interactions and ultimately to influence patients’ well-being. We assume that improvements in well-being are associated with high-quality care delivery as well as cognitions and behaviors of older people and healthcare professionals. The proposed concepts and their interrelations are explained in detail hereafter.

### Quality of integrated primary care for frail community-dwelling older people

In order to effectively redesign primary healthcare for frail community-living older people, it is important to consider promising components of successful innovative primary care approaches aimed at supporting their needs to realize well-being. An overall state of well-being is determined by an older person’s ability to achieve universal goals of social and physical well-being that are, in turn, achieved through five instrumental goals (stimulation and comfort for physical well-being and status, behavioral confirmation, and affection for social well-being) [32–34]. Integrated care in the primary care setting is expected to support these needs and therefore improves or protects well-being [19]. Earlier research already showed that quality of care affected the well-being of community-dwelling COPD patients [35]. Integrated care is defined as ‘a well planned and well organized set of services and care processes, targeted at the multi-dimensional needs/problems of an individual client, or a category of people with similar needs/problems’ [36]. The World Health Organization, for example, stated that by introducing integrated care, health services will be more responsive to frail older people’s needs [37]. A systematic review of Eklund and Wilhelmson [28] indeed provided some evidence regarding the benefits of integrated care for frail community-dwelling older people. In general, these integrated care approaches consist of multiple interrelated components, such as proactive case finding, case management, medication review, self-management support, and working in multidisciplinary teams [27–30]. Efforts to improve primary care for frail older people should integrate these promising interrelated components in order to assure that activated, informed older adults can productively interact with prepared, proactive healthcare professionals of primary care teams [38]. Still, we lack understanding of the underlying mechanisms that explain how integrated primary care delivery affects outcomes. Earlier research investigating mechanisms explaining the effectiveness of integrated care showed that cognitive and behavioral components of healthcare professionals and older patients drive effectiveness in terms of productive patient-professional interactions and well-being [39–44].

### Cognitive and behavioral components

#### Productive patient-professional interaction

Well-designed healthcare systems should be able to meet the needs and preferences of frail community-dwelling older people by means of fostering productive interactions between these older patients and their (team of) healthcare professionals [45, 46]. These productive interactions are at the core of patient-centered care [47]. They are considered important in achieving the best possible patient outcomes [37, 38, 45], like well-being [46]. Productive patient-professional interactions are characterized by reciprocal interrelations between professionals and patients and high levels of shared goals, communal knowledge, and mutual respect [48–50]. Such productive patient-professional interactions were indeed associated with enhanced well-being of patients [51].

Hereafter we conceptualize the proposed underlying cognitive and behavioral mechanisms explaining effectiveness of integrated primary care approaches. These cognitions and behaviors of healthcare professionals and older adults are presumed to have a direct association with patients’ well-being. In addition, cognitions and behaviors are believed to foster productive patient-professional interactions which, in turn, impact well-being of frail older patients.

#### Cognitions and behaviors of frail older people

Individuals take an active role in realizing well-being and aim to enhance their life situation by optimizing the universal goals of physical and social well-being [52–56]. Frail older people often experience a decline in reserves and resources in multiple domains, e.g. health status, loss of mobility, cognitive functioning, and social activities. This implies that well-being of older people in particular is more likely to be negatively affected by decaying reserve-capacities that otherwise may compensate sufficiently for these losses in resources. Their cognitions and behaviors may foster (or hamper) productive patient-professional interactions and allow them to regulate their resources and cope with or avoid losses in order to protect their well-being [57]. Moreover, the degree to which chronic conditions are controlled and outcomes are achieved depends partly on the effectiveness of frail older people’s behavioral and cognitive self-management abilities. It is therefore considered essential to involve patients in their own care process [58]. Empowered patients that are effective self-managers are better equipped to control chronic conditions and to positively influence outcomes [59]. Key cognitive and behavioral abilities for managing resources for well-being identified earlier are (i) taking initiatives, (ii) investing in resources for benefits in the longer-term, (iii) maintaining a variety in resources, (iv) warranting multifunctionality of resources, (v) self-efficaciously managing resources, and (vi) keeping a positive frame of mind [57, 60]. These identified key self-management abilities include relevant cognitions, i.e. self-efficacy beliefs and a positive frame of mind, which advance the ability to take action. These cognitive processes are essential for both coping with losses and (pro)actively managing resources. A positive frame of mind refers to the ability to maintain positive expectations for the future, even in adversity. Self-efficacy beliefs, i.e. the belief in one’s own ability to successfully interact with the environment and pursue goals, are important for the performance of many behaviors [57]. For example, low self-efficacy can lead people to believe they lack the ability to effectively perform a certain behavior that brings desired outcomes, which in turn may result in not engaging in that behavior [61]. At later stages of life, self-efficacy beliefs may be declined by, for example, physical disabilities and experiences of loss [57]. These cognitions are relevant but not sufficient. Although a person may have a strong sense of efficacy, he or she needs to perform the particular behavior to achieve desired outcomes. Therefore, Steverink and colleagues [57] underline the importance of active-motivational processes with respect to managing resources, i.e. taking initiative and investment behavior [57]. As a result of a decline in reserves and resources, there may be a loss of autonomy and an increase in dependency in old age [62]. It is suggested that taking the initiative regarding relevant resources in contrast to being passive or dependent is important to attain or maintain well-being. Moreover, investment behavior is assumed to be important in realizing or maintaining well-being as investing in key resources is considered relevant for stability in resources. In addition to cognitions and active-motivational processes, resource-combining processes are presumed relevant, which include realizing multifunctionality of resources and a variety in resources [57]. Important for realizing well-being are resources that meet various dimensions of well-being at the same time in a mutually reinforcing way, for example, activities serving both social and physical well-being [63, 64]. In addition, a variety in resources is assumed to be of importance and refers to having multiple resources to realize a particular aspect of well-being. Resource-combining processes can create buffers against a loss of well-being [64]. Thus, these key cognitive and behavioral abilities are considered most essential in managing losses adequately and managing resources effectively to realize, maintain or improve well-being [57]. In addition to this, strengthening cognitive and behavioral abilities among frail older people is expected to lead to more productive patient-professional interaction, which in turn is expected to improve the well-being of frail older people [46, 51]. For productive patient-professional interaction to occur, patients need to be informed (equipped with adequate information in order to become proactive partners and effective decision makers in the care process) and activated (understanding the significance of sharing information and the importance of their own role in the care process) [51].

#### Cognitions and behaviors of healthcare professionals

In addition to the behaviors and cognitions of frail older people, the behaviors and cognitions of healthcare professionals also drive effectiveness of integrated care approaches [39–41, 43]. It is therefore crucial to gain insight into the cognitions and behaviors of individual healthcare professionals. According to Salas and colleagues [65], individual professionals need to have the right knowledge (cognitions) and skills (behaviors) [65]. Cognitive components reflect the mechanisms that change the way individual healthcare professionals think [39]. We focus on the concept of situation awareness as it is considered a central construct for decision making and performing actions in complex, dynamic systems like healthcare [66]. Situation awareness is defined by Endsley [67] as a person’s awareness of the elements in the environment (perception), understanding of the significance of those elements (comprehension), and ability to project future actions to allow timely decision making (projection); or simply “knowing what is going on”. It comprises a person’s state of knowledge about the environment [67] and can be thought of as an internal mental model of the present environment of a healthcare professional. These mental models allow people to interact effectively with their environment [66, 68]. Healthcare professionals need to synthesize all incoming data from, among others, information systems, communications (e.g. individualized care plan), patients, and fellow professionals. This results in an integrated representation of the current status of the patient. In the work process, healthcare professionals are involved in developing and updating situation awareness in a complex and changing work environment [69]. To allow professionals to effectively respond to the needs of the patients, professionals need to perceive the critical factors in the current situation of a patient (e.g. being aware of chronic conditions and levels of frailty), understand the meaning of those factors (e.g. integrate information on present chronic conditions and different treatments options) and project future actions (e.g. predict the response of a patient to a certain treatment) [69–71]. Quality of care and frail patients’ outcomes are therefore dependent on the professionals’ knowledge and understanding of the patient’s current situation. In addition to situation awareness, cognitive diversity has also been identified as underlying mechanism explaining effectiveness of integrated care programs [39]. Cognitive diversity refers to differences in knowledge, beliefs, preferences, and perspectives among professionals [72]. The integration of this diversity in cognitions, which mirrors the knowledge and skills of various disciplines, is related to the development of new knowledge among each team member [72, 73]. Especially in the case of complex patient populations, such as frail community-dwelling older people, patients are expected to benefit from a wide range of skills and different types of knowledge [74]. In addition to these cognitions, behaviors such as collaboration and coordination among healthcare professionals with different areas of expertise are also essential [21, 29, 38]. Coordination can occur through a structure of relational and communicational links among multiple professionals in a work process which consists of interdependent tasks. It involves managing interdependency of tasks as well as interdependency of professionals that execute the tasks [75]. For coordination to be effective, the quality of communication (e.g. frequent communication) among individual professionals is important. The quality of communication depends on the quality of underlying relationships (e.g. mutual respect) among healthcare professionals. Inversely, the quality of relationships is dependent on the quality of communication. This is known as relational coordination [48].

Above-mentioned cognitive and behavioral components among patients and professionals are assumed to be important in fostering productive patient-professional interactions and improving well-being of frail older patients. Based on the literature, we presume that patients’ and professionals’ behaviors and cognitions are the underlying mechanisms explaining effectiveness of integrated care. The use of integrated care components such as proactive case finding, case management, and medication review are, for example, known to be more effective among teams with high-quality interactions and collaboration among professionals of different disciplines [76]. Diverse healthcare professionals must be strongly connected for integrated primary care approaches to provide effective care [77]. In addition, self-management support is more effective among frail older patients with cognitions and behaviors that foster productive patient-professional interaction, allowing them to effectively regulate their resources and improve their well-being [57]. Therefore, patients’ and professionals’ behaviors and cognitions should be investigated when the effectiveness of integrated primary care approaches for frail community-dwelling older people is evaluated. This may help to increase our understanding of the (inconclusive) effects of integrated primary care approaches and underlying mechanisms explaining their (lack of) effectiveness.

### A theory-guided study protocol to evaluate the integrated primary care approach “Finding and Follow-up of Frail older persons”

#### Description of the “Finding and Follow-up of Frail older persons” approach

The theory-guided study protocol is based on an integrated primary care approach called "Finding and Follow-up of Frail older persons" (FFF). The FFF approach combines promising components of integrated primary care, including proactive case finding, case management, medication review, self-management support, and working in multidisciplinary teams. The FFF approach is implemented in several GP practices in the western part of the Province of North Brabant in the Netherlands and aims to target frail community-living older people. The main objectives of the FFF approach are: (1) establishment of a proactive integrated primary care system for frail community-dwelling older people (consisting of collaboration among professionals with different occupational backgrounds led by a GP), (2) avoidance of hospital and nursing home (re-)admissions, and (3) improvement of well-being and self-management abilities. The integrated primary care approach advocates a proactive primary care practice team in which the GP has the lead. The multidisciplinary setting enables the development of the role of the elderly care physician and geriatric nurse within the primary care setting. An elderly care physician is a primary care expert in geriatric medicine and is specialized in long-term care for frail older patients with complex needs [78, 79]. The Netherlands is a trendsetter with respect to training physicians for this specific group of patients in a primary care setting [79]. In more detail, the following key elements of proactive integrated primary care are incorporated in the FFF approach.

##### Proactive case finding

With the aging of the population, an increasing trend in frailty is to be expected. Case finding of frail independently living older adults becomes of major importance and it is suggested that all older people should be screened for frailty by healthcare providers [80]. Especially the primary care setting is considered suitable for proactive case finding as it is stated that 80 percent of all frail community-living older people consulted their GP in the past three months [81]. In order to find potentially frail older people in the community, the GP selects older people based on, for example, gut feeling, i.e. a ‘sense of alarm’. These selected older patients are then visited at home by the geriatric nurse or practice nurse and screened for frailty by means of the Tilburg Frailty Indicator (TFI). The TFI is a 15-item questionnaire that assesses frailty in the physical, psychological, and social domain [82]. This instrument was developed based on the definition of frailty as stated by Gobbens and colleagues [6], namely ‘Frailty is a dynamic state affecting an individual who experiences losses in one or more domains of human functioning (physical, psychological, social), which is caused by the influence of a range of variables and which increases the risk of adverse outcomes’ [6]. Scores on the TFI range from 0 to 15 and older patients with a TFI score ≥ 5 are identified as frail [82]. Moreover, the practice nurse or geriatric nurse will perform physical measures or additional interviews with the older person when necessary (e.g. Mini–Mental State Examination (MMSE) to assess cognitive functioning). Hence, it may happen that a person is not frail according to the TFI (score ≤ 4) but is considered frail based on examination of the nurse. We consider these additional interviews important as the TFI may not grasp all relevant aspects of frailty and hence it is recommended not to use the instrument in isolation [83].

##### Case management

Case management is expected to improve quality of primary care for frail community-dwelling older people as well as delay or avoid institutionalization. The case manager in the FFF approach is expected to support the provision of proactive integrated care through a collaborative process of assessment, planning, facilitation, care coordination, evaluation, and advocacy for options and services to meet frail older patients’ needs [84]. The FFF approach uses home visits by case managers to achieve these goals. Furthermore, the case manager acts as a boundary spanner to ensure a well-functioning team of professionals supporting frail older patients.

##### Medication review

Older persons’ medicines are systematically and critically examined in a medication review. An important aspect of multidisciplinary consultation is the assessment of prescribed and over-the-counter medications used by these older people. The most recent overview of medications used by the older person, and experiences with medications, are discussed with the person (and informal caregivers or relatives). Possible additional actions include: (i) visitation of the older person by the elderly care physician to provide additional information about medications, (ii) the GP’s discussion of the person’s case history with the pharmacist, and (iii) the establishment of agreement about medication use between the GP and second-line medical care.

##### Self-management support

The FFF approach aims to improve self-management abilities and well-being among frail patients by incorporating different types of self-management support interventions, like skill building, educational materials, personal coaching, and the use of an individualized care plan. Needs and problems are listed by means of the so called SFSPC-model of reporting on Somatic, Functional, Social, Psychological, and Communicative indications for each individual frail older person. Subsequently, the individualized care plan is established and recorded, including the problems and needs, the formulated goals, and the possible actions and interventions. Agreements are made regarding follow-up and patients’ cases are evaluated at least once a year. Specific protocols for patient referral are established. For example, older persons are asked to identify preferred healthcare organizations and professionals (e.g. physiotherapists) in the fields of care and welfare. These preferred professionals are approached by the GP, elderly care physician or practice nurse. The professionals provide feedback information about patient care to the GP and/or elderly care physician.

##### Multidisciplinary teams

A strong team of professionals with different occupational backgrounds led by a GP is one of the core elements of the FFF approach in order to deliver high-quality care to frail elderly patients. Each case of an older person is discussed in multidisciplinary consultation. An inventory of relevant healthcare professionals is made by the GP and/or case manager and these professionals are invited to attend the consultation. They involve professionals in the care and treatment of patients (e.g. elderly care physician, physiotherapist, and psychologist) as well as professionals in the field of welfare when necessary. In the FFF approach, the elderly care physician plays an important role in the care process for older persons. Next to being present at the multidisciplinary consultations, the GP can obtain advice from the team’s elderly care physician on several complex health problems, e.g. depression and apathy, somatic or geriatric indications, and problem analysis in case of multimorbidity. The GP and the elderly care physician discuss whether one or several consultations are needed to assess each older person’s relevant healthcare needs. When necessary, other health and social care professionals (e.g. palliative care nurse) are involved. Plans and actions that are discussed during the multidisciplinary consultations are then discussed with the patient, tailored to the patient’s needs and wishes, and reported in the individualized care plan.

The interrelated key elements of the FFF approach are combined in a comprehensive approach to provide integrated primary care that can be tailored to the wishes and complex healthcare needs of frail community-living older people. The elements of the FFF approach are based on promising components of integrated care that were found in the literature [27–30]. As was explained in the previous section, we presume that this multicomponent approach influences the cognitions and behaviors of older patients and healthcare professionals which ultimately are expected to affect the productivity of patient-professional interactions and older patients’ well-being (See Fig. 1 and section ‘A theoretical model to facilitate the evaluation of integrated primary care approaches’).

## Methods

The second aim of our study is the development of a theory-guided study protocol to evaluate the innovative primary care approach FFF aimed at improving well-being among frail community-dwelling older people. We will explain the proposed methods to be used for our theory-guided evaluation of the FFF approach.

We aim to investigate (i) the potential effectiveness of the FFF approach in improving well-being among frail community-dwelling older people (*effect evaluation*), (ii) the implementation and context of the FFF approach in order to facilitate the interpretation of the results (*process evaluation*), and (iii) the cost-effectiveness of the FFF integrated primary care approach (*economic evaluation*).

### Study design

The longitudinal evaluation study has a mixed methods design in which a combination of quantitative and qualitative research methods are employed in order to evaluate the effectiveness, processes, and cost-effectiveness of the FFF approach. The evaluation study is performed between 2014 and 2017. The study has a matched quasi-experimental design with one pretest and one posttest measurement, i.e. the effects are measured before and after the intervention. Measurements are performed at baseline (T0) and 12 months thereafter (T1). Moreover, the study includes an intervention and a control group (i.e. intervention and control GP practices).

### Ethics approval

The research proposal has been reviewed by the medical ethics committee of the Erasmus Medical Centre in Rotterdam, the Netherlands (study protocol number MEC-2014-444). The committee decided that the rules laid down in the Medical Research Involving Human Subjects Act did not apply.

### Setting and GP practices

The study is performed in the western part of the Province of North Brabant in the Netherlands. This region contains a relatively high proportion of frail older persons compared with many other regions in the Netherlands [85]. GP practices in the region were eligible to participate in the intervention group of the study if they were not involved in other research projects and had implemented the FFF approach recently. Control GP practices were eligible for participation if GPs were not engaged in or planning to start screening older adults on frailty. In addition, GP practices that already follow-up older persons in a systematic way were not eligible to participate as control GP practices. Control GP practices continue to provide usual primary care and patients are able to use all available (primary care) services as before. We approached 17 GP practices for participation in this study (12 intervention practices and 5 control practices). In total, 11 of 12 GP practices that recently implemented the FFF approach agreed to participate in the study and 4 of 5 control GP practices consented to participate. The reasons for non-participation (2 GP practices) were the workload and time constraints. GP practices receive a small financial compensation for the administrative burden associated with the evaluation study.

### Participants and recruitment of frail older people for the FFF approach

The target population of the study consists of community-dwelling older persons aged 75 years and older registered at the 15 participating GP practices. With increasing age, the prevalence of frailty increases substantially [4, 86, 87]. Therefore, we decided to include persons aged 75 years and older. A four-stepped approach is used to describe the study population in terms of frailty, select patients that are eligible to participate in the FFF approach, include eligible patients in the evaluation study and match patients of intervention GP practices to patients in the control group (one-to-one matching).

***Step 1:*** Frailty is assessed among patients aged 75 years and older registered at the 15 participating GP practices. All older patients receive the validated TFI to screen for frailty [82]. Next to the TFI questionnaire, we provide a letter on behalf of the GPs and researchers, an information leaflet about the study, and a postage free return envelope. After 2-3 weeks, reminders are send to non-responders by mail and/or older patients are reminded by means of a telephone call. Older patients with a TFI score ≥ 5 are identified as frail [82]. The aim of this inventory is twofold, namely (i) to assess frailty in a community-dwelling population of older persons, and (ii) to arrive at frailty scores for the one-to-one matching procedure (Step 4) of patients that are selected and eligible to participate in the evaluation study.

***Step 2:*** The TFI frailty scores of older patients are handed over to the participating GPs in order to provide insight into their older patient population.

***Step 3:*** GPs of the intervention group make their own selection of eligible patients to be included in the FFF approach. This selection can be based on the frailty scores obtained by the administration of the self-report TFI questionnaire but can also be based on additional interviews and measures that are performed by the healthcare professional as part of the care provision. Although the TFI is a reliable and valid instrument for measuring frailty in community-living older adults [82, 88], the previously stated added value of other interviews and measures to assess frailty underlines the importance of not relying solely on the TFI as a measure to identify frail older patients [83]. Consequently, patients that are not frail according to the TFI (score ≤ 4) but are considered frail based on other examinations performed by healthcare professionals may nevertheless be selected for the FFF approach.

***Step 4:*** Eligibility of the frail older people selected in Step 3 is then assessed in terms of the inclusion and exclusion criteria by the researchers. For participation in the evaluation study we exclude (1) older people living in nursing homes or homes for the elderly, (2) persons having an estimated life expectancy of less than 3 months, and (3) people with an inadequate understanding of the Dutch language. A challenge in quasi-experimental designs is to reduce the risk of selection bias, i.e. preexisting differences in characteristics between the intervention group and control group due to the absence of random intervention assignment. This may result in a biased posttest measurement. In order to acquire unbiased estimates of the effects, the most important covariables should be balanced between intervention and control groups [89, 90]. To increase comparability of the intervention and control groups, we use one-to-one matching: each individual participant in the intervention group is matched to one participant in the control group with the same values of the key covariables, namely sex (male or female), frailty score (score on the TFI), and educational level (high or low). This one-to-one matching is performed by the researchers.

In total, 500 frail older patients are included (250 patients in the intervention group and 250 patients in the control group). Next to frail older patients, we include healthcare professionals in our evaluation study. All healthcare professionals involved in the healthcare delivery for older patients are approached to participate in our study. Our aim is to guarantee the inclusion of healthcare professionals with various backgrounds and areas of expertise, e.g. GPs, elderly care physicians, physiotherapists, case managers, practice nurses, and social workers. Moreover, we recruit professionals involved in the management of integrated care delivery. Approximately 60 professionals in the intervention group and 60 professionals in the control group are included.

### Healthcare delivery: Intervention group and control group

Frail older persons in the intervention group receive the proactive, integrated care approach FFF as was previously described in detail. Frail older people in the control group receive usual care services available for older people as arranged by their GP practice and local health and community organizations.

### Data collection and informed consent for the evaluation study

Older persons in the intervention group and control group are interviewed at home at baseline (T0) and 12 months thereafter (T1). Interviewers are recruited in the western part of the Province of North Brabant in the Netherlands to assure a cultural fit with the older persons and all interviewers have a background in healthcare. Interviewers are trained to conduct the interviews and are blinded to the status of the older patients, i.e. patient of an intervention GP practice or control GP practice. Before contacting potential eligible older persons to participate in the study, the GP assesses whether reasonable grounds to suspect incapacity to either participate in the study or to give consent due to cognitive impairment exist (based on their medical records and latest encounters with the GP). In case of doubt the GP will contact the older person’s informal caregiver (spouse or children) to discuss the patient’s current (cognitive) state which will lead to the GP’s final assessment. Those who are considered incapable by the GP will be excluded from the study. This procedure will be followed at both T0 and T1. Eligible older patients are then informed by telephone and during the home visit about the study (verbal explanation of the study purposes, procedures, confidentiality, and contact information). In addition, patients receive a leaflet with research information. It is explicitly stated to patients that their voluntary participation in the evaluation study does not affect healthcare delivery. Patients registered at intervention GP practices can participate in the FFF approach even though they are not willing to participate in the evaluation study. Patients that are willing to participate in the evaluation study are interviewed after they sign an informed consent form. The informed consent form states that the patient may discontinue participation in the study at any time without adverse consequences or loss of benefits. On average, the duration of an interview is 60-75 minutes. Outcome data and demographic data from healthcare professionals are also collected at baseline (T0) and 12 months thereafter (T1) (see section ‘Outcome measures and measurement instruments’). Postal self-report questionnaires are used to collect data among the professionals involved in the healthcare delivery for frail independently living older patients. After 2-3 weeks, reminders are send to non-responders by mail and/or healthcare professionals are reminded by means of a telephone call.

### Outcome measures and measurement instruments

To assess the effectiveness of the FFF integrated primary care approach in improving well-being of frail community-living older patients, we selected measurement instruments that are particularly relevant for measuring all the concepts incorporated in our proposed theoretical model. All outcomes are measured at baseline (T0) and 12 months thereafter (T1). Outcome measures for the economic evaluation are described in the section ‘Economic evaluation’.

### Primary outcome measure

#### Well-being

To measure individuals’ realization of universal goals needed to enhance their well-being, the 15-item Social Production Function Instrument for the Level of well-being (SPF-IL) is used [34]. Social Production Function (SPF) theory, as introduced by Lindenberg [52–54], asserts that five instrumental goals, i.e. comfort, stimulation, status, behavioral confirmation, and affection, are important for optimizing the universal goals of physical and social well-being [32, 33]. This instrument has been thoroughly validated by Nieboer and colleagues [34] and is used frequently among (frail) older populations (e.g. [44, 51]).

### Secondary outcome measures

#### Cognitive and behavioral components

##### Productive patient-professional interaction

To assess productive patient-professional interactions, we measure dimensions of communication and relationships among community-living frail older persons and their healthcare professionals using a validated relational coproduction instrument. Relational coproduction will be measured by means of 7 survey questions. Frequency, timeliness, accuracy, and problem-solving nature of communication as well as quality of the relationships are measured. The latter aspect focuses on mutual respect and the extent to which goals and knowledge are shared. Frail older patients are asked to assess the quality of their communication and relationships with the healthcare professionals involved in their care process (e.g. GPs, practice nurses, physiotherapists). Similarly, healthcare professionals assess the quality of the communication and relationships with patients. Together these dimensions form the relational coproduction construct [76, 91–93].

##### Cognitions and behaviors of frail older people

Cognitive and behavioral self-management abilities are measured by means of the short version of the Self-Management Ability Scale (SMAS-S). The SMAS-S contains 18 items assessing six core cognitive and behavioral abilities of self-management, i.e. self-efficacy beliefs, a positive frame of mind, taking initiative, investment behavior, multifunctionality of resources, and variety in resources [57, 60, 94]. This instrument has also been thoroughly validated among older populations by Cramm and colleagues [94].

##### Cognitions and behaviors of healthcare professionals

Dimensions of communication and relationships among healthcare professionals (i.e. relational coordination) are measured similarly to the assessment of relational coproduction in older people and their healthcare professionals. The 7 questions of the validated measure of relational coordination assess the dimensions of communication (frequency, timeliness, accuracy, and problem-solving nature of communication) and relationships (shared knowledge, shared goals, and mutual respect) among healthcare professionals [95]. Professionals involved in healthcare delivery for frail older people (e.g. GPs) are asked to assess the quality of their communication and relationships with other professionals (e.g. elderly care physicians). As a result, we evaluate separately the connections of healthcare professionals with other types of professionals involved in the care process. Altogether these dimensions form the construct of relational coordination [76, 91–93]. We followed earlier research that also used this instrument to assess cognitions and behaviors among professionals [40, 41].

### Quality of integrated primary care for frail community-dwelling older people

#### Older patients’ experiences with integrated primary care

Quality of integrated care is measured using the short version of the Patient Assessment of Chronic Illness Care (PACIC-S) [96]. The 11-item PACIC-S measures the extent to which care is proactive, planned, and patient-centered as perceived by patients [38, 97, 98]. In addition, the instrument incorporates key components related to self-management support, e.g. goal setting, problem-solving, and follow-up [99, 100].

#### Healthcare professionals’ perceptions of integrated primary care

Quality of integrated care as perceived by healthcare professionals is assessed by means of the 21-item short version of the Assessment of Chronic Illness Care (ACIC-S) [101]. The instrument comprises six levels of system change that affect quality of healthcare delivery [102].

### Covariables

Several variables will be measured to provide insight into the characteristics of the study population and to facilitate interpretation of the outcomes of the evaluation study. Socio-demographic data (e.g. age, sex, marital status, educational level, net household income, and living situation) are collected during the interviews with frail older patients. We assess several additional variables in order to attempt to account for potential case-mix differences that may be introduced due to the non-random allocation of older patients to the intervention and control groups. Multimorbidity, physical functioning, cognitive functioning, and social functioning are assessed. Morbidity is assessed by indicating morbidities experienced in the past 12 months from a predefined list of 17 conditions (e.g. diabetes, chronic obstructive pulmonary disease (COPD), osteoporosis, and cancer). Multimorbidity is defined as the presence of two or more conditions from this list [103]. Physical functioning is assessed by means of a modified version of the Katz Activities of Daily Living index (Katz ADL index). Functional limitations are assessed for 8 activities in daily life, i.e. bathing, dressing, toileting, eating, continence, transfer, walking, shaving or to comb one’s hair [104, 105]. Additionally, cognitive functioning is assessed by means of the 12-item Mini-Mental State Examination (MMSE-12). The MMSE-12 focuses on cognitive aspects of mental functions and includes elements like orientation to time and place, recall of words, and complex commands, e.g. drawing a figure [106, 107]. Social functioning is assessed by means of the social component of the Dutch version of the 20-item Short Form Health Survey (SF-20) [108–110]. Next to the additional variables that are measured at the level of the older adults, we collect socio-demographic data of healthcare professionals (e.g. age, sex, educational level, occupation, and working hours).

### Process evaluation

An integral part of the evaluation of the FFF approach to determine the quality of integrated care and to understand underlying mechanisms explaining effectiveness is the process evaluation. A process evaluation is useful because of the complexity of the integrated care approach, which comprises multiple elements and affects various outcomes [111]. These elements may be mutually reinforcing and have a synergistic effect [112]. Also, GP practices differ with respect to important characteristics. Oakley and colleagues [113] state that approaches may be implemented and received differently across sites. According to Øvretveit and Gustafson [112], effectiveness of integrated care approaches often depends on the degree of implementation. Assessment of the implementation and context is therefore essential and may help to gain insight in how processes work to produce effects. Thus, it is important to describe the multicomponent approach, its implementation and context, and discover the factors that are crucial for the implementation and outcomes [112]. Therefore, the aim of the process evaluation is to provide a thorough description of the FFF approach, assess the implementation and the context of this integrated care approach, and to provide factors and conditions that are critical for success. This also applies to the control practices. They will be studied in-depth and usual care delivery will be richly described. The process evaluation study identifies whether the FFF approach contributes to integrated care delivery in order to effectively support independently living older persons. We aim to enhance our understanding of the challenges faced by healthcare organizations and professionals and identify factors that facilitate or hinder the implementation of integrated care in a primary care setting. Therefore, in addition to the quantitative data described in the previous section, also qualitative data are gathered from actors at all levels, including frail older persons, healthcare professionals involved in the care process, and professionals involved in the management of healthcare delivery. A combination of quantitative and qualitative data will provide a richer understanding of the effects and processes of integrated primary care for frail older people.

Process indicators are registered continually during the follow-up period of 12 months. Descriptions of patient visits by practice nurses and geriatric nurses, medication reviews, assessment outcomes, and individualized care plans describing the provided self-management support are analyzed. Data are collected from data registries (e.g. information systems). We collect data on several process indicators that are specifically related to the FFF approach. Examples are the number of frail older patients that are discussed in multidisciplinary consultations, the number of older adults that have an individualized care plan, and the number of multidisciplinary consultations per year. Moreover, field notes are made at several multidisciplinary consultations executed by intervention GP practices and general meetings related to the FFF approach. Examples are educational meetings for geriatric nurses and practice nurses, steering committees, and working groups. Furthermore, semi-structured face-to-face interviews are conducted with a sample of healthcare professionals involved in the care process for frail independently living older patients. We aim to interview a diverse group of professionals, including practice nurses, elderly care physicians, and case managers. Hence a purposeful sampling procedure will be used, which can be used in qualitative research to collect data from the participants who are knowledgeable about the topic [114, 115]. Healthcare professionals are encouraged to describe and reflect on their experiences with healthcare delivery for frail older patients living in the community. In addition, several professionals involved in the management of healthcare delivery are interviewed. Examples are the project manager of the FFF approach and policy advisors of different healthcare organizations (e.g. homecare agencies). Also, semi-structured face-to-face interviews are held with a sample of independently living frail older persons. The aim is to gather the reflections on experiences of patients with the FFF approach (intervention group) or care as usual (control group) from their own perspective. To create a diverse sample of older people, we aim to select participants with different characteristics. Besides these semi-structured interviews, face-to-face interviews based on a more structured interview template are held with GPs of all participating GP practices as well. As the GP has the lead in the implementation and execution of the FFF integrated care approach, we decided to interview GPs to assess exactly how care is being delivered in the different intervention GP practices. We also assess how care for community-dwelling older people is being delivered in the control GP practices (care as usual). We developed an interview template based on the Chronic Care Model (CCM) [38, 97]. The CCM highlights system changes in several areas (e.g. decision support and clinical information systems) to guide quality improvements [38, 97]. Important interventions related to care delivery are classified according to the areas of system change in the interview format. Examples of interventions are the systematic follow-up of patients and the use of clinical guidelines. Data about the implementation of these various interventions within each of the areas of system redesign are collected. Altogether an extensive description of (un)successfully implemented interventions is provided. Interviews are recorded with permission of the GPs and finalized data are send back for member checking and corrections.

### Qualitative analyses

All interviews are audio-taped with permission of the patient or professional. After the transcription of the audio-taped interviews, latent content analysis is used [116, 117] in which the focus is primarily on analyzing the underlying meaning of the content [118, 119]. The Dutch texts derived from the interviews will be translated into English. All texts will be read several times by researchers with expertise in qualitative research to ensure a holistic understanding. We will extract, code, and categorize meaning units. Underlying meanings of categories will be expressed in themes [116].

### Integration of qualitative and quantitative data

An embedded mixed methods design is used where qualitative data is added to the quasi-experimental design. During the study both quantitative and qualitative data will be collected and analyzed. For example, qualitative data will be collected during the study to explore how participants experience the FFF approach. By using this embedded design the qualitative data augment the (cost)effectiveness study, which is a popular approach within implementation and dissemination research similar to the FFF approach [120, 121]. We will also use an explanatory design in which qualitative data helps to understand quantitative results [115]. We will for example collect and analyze quantitative data with regard to the quality of (integrated) care as perceived by healthcare professionals. Subsequently, these results will be followed up with in-depth qualitative data to explain and expand the quantitative results (e.g. unexpected results, significant differences between the groups in perceived quality of care).

### Economic evaluation

The healthcare expenditures have increased substantially in the previous decades [122]. Moreover, an increase in healthcare costs associated with aging is expected [123]. In response to these increases, challenges with respect to allocating scarce healthcare resources over interventions become apparent [124, 125]. The decision to fund and implement one intervention over another will not only have an effect on patient outcomes, but also on (publicly funded) healthcare resources and resources outside healthcare. Accordingly, consideration of effects as well as costs is important in informing decisions related to the optimal resource allocation and reimbursement of healthcare approaches. Such decisions require evaluations that go beyond assessing merely effectiveness and processes of healthcare approaches, and should include an economic evaluation as well [125].

The primary aim of our economic evaluation is to determine whether the FFF approach is cost-effective when compared to care as usual for frail community-dwelling older persons. We comparatively assess the costs and effects of the FFF integrated primary care approach and care as usual. The economic evaluation is performed from the societal perspective which implies that, in principle, all relevant costs and effects are incorporated in the analysis [125, 126]. Our economic evaluation comprises a cost-utility analysis and a cost-effectiveness analysis.

For the cost-utility analysis, the effectiveness is measured using quality-adjusted life-years (QALYs). QALYs are a preference-based health measure that comprises length and health-related quality of life [127]. For the measurement of an older person’s health-related quality of life, we use the EuroQol (EQ-5D-3L) health-related quality of life instrument that covers 5 health dimensions: mobility, self-care, usual activities, pain and discomfort, and anxiety and depression. Scores on a three-point scale indicate the older person’s level of functioning: no problems (1), some or moderate problems (2) or severe problems (3) [128, 129]. Utility scores are calculated by means of the Dutch tariff [130]. The utility scores are used to calculate QALYs gained or lost during the follow-up period of the study by applying the area-under-the-curve method [131, 132]. Estimating QALYs allows the comparison of our outcomes with the cost-effectiveness of other integrated primary care approaches [133].

In economic evaluations of healthcare approaches that are aimed at improving well-being, it is recommended to use well-being instruments alongside the more conventional health-related quality of life instruments [134]. QALYs focus mainly on health and to a lesser degree on well-being in general [135]. With respect to evaluating primary care approaches aimed at frail older people, it is crucial to have outcome measures that also go beyond health and evaluate a wider range of benefits for older people [136]. Due to the lack of preference scores or utilities for well-being, a cost-effectiveness analysis is conducted with incremental effectiveness expressed as the difference in mean SPF-IL scores. Scores on the four-point scale of the multidimensional SPF-IL instrument range from never (1) to always (4). Higher mean scores indicate a greater well-being [34]. Both the SPF-IL and the EuroQol (EQ-5D-3L) are completed by frail older patients at baseline (T0) and 12 month follow-up (T1).

Intervention costs, healthcare costs, and patient-related costs are considered relevant for the economic evaluation. *Intervention costs* consist of all costs that can be attributed to the delivery of the FFF approach, excluding the research-specific costs (e.g. executing the interviews with older patients and professionals). Examples are costs that are associated with the proactive case finding of frail elderly in the community and multidisciplinary consultations in GP practices. We assess the average amount of time for each of the activities related to the FFF approach using time registrations of healthcare professionals and by registering time during observations. *Healthcare costs* relate to (telephone) consultations with GPs and practice nurses, emergency GP, admissions to hospitals, nursing homes or homes for the elderly, homecare services, day care, nursing care, visits to paramedics, psychosocial care, and prescribed medications. Only consultations that are not already part of the FFF integrated care approach are included. Healthcare utilization is assessed by means of extracting data from electronic health records within GP practices and homecare organizations. Moreover, health service use is measured by asking older adults directly about their healthcare use during the interview (see paragraph ‘Data collection and informed consent for the evaluation study’). Patients indicate at baseline (T0) and 12 month follow-up (T1) what type of care they received and how often (e.g. hours of homecare or days of hospitalization). *Patient-related costs* include costs that are covered by frail older people themselves, like purchasing assistive aids (e.g. wheeled walkers) or time investments related to the FFF approach. This data is collected during the interviews with older persons.

For the valuation of the healthcare costs, the latest version of the Dutch manual for costing in healthcare is used [133]. We multiply volumes of resource use (e.g. days in hospital) with standardized costs per unit of resource use (in euros) to estimate costs of each approach (i.e. FFF approach and care as usual). When these standardized costs per unit of resource use are not available, costs are estimated using true economic costs, average reimbursement fees or literature.

The economic evaluation includes calculations of the cost-effectiveness and cost-utility ratios. For comparing the costs and effectiveness of the FFF approach and usual care, incremental cost-effectiveness ratios are calculated (ICERs). In this way, the additional costs and effects of the FFF approach compared with usual care are determined. The ICER in the cost-effectiveness analysis represents the incremental costs per point improvement in well-being (SPF-IL score). The ICER in the cost-utility analysis expresses the incremental costs per QALY gained. In the ratio, the numerator includes the difference in costs and the denominator the difference in effects [124]. Sensitivity analysis is performed to assess the robustness of a series of predefined assumptions. A cost-effectiveness plane and an acceptability curve are added.

### Sample size

We aim to include 500 frail older patients (250 patients of intervention GP practices and 250 patients of control GP practices). We aim to optimize participation by means of personal home visits, however, we anticipate a drop-out rate of approximately 20% between T0 and T1 (e.g. due to death, refusal). Accordingly, we expect 400 patients at T1 in the intervention and control groups. Sample size calculations are based on the mean well-being score (SPF-IL) of a comparable Dutch population of frail community-dwelling older persons (N=945) [137]. To detect a mean improvement in well-being of 1/4 standard deviation (SD) based on the SPF-IL, we need at least 198 older patients in each group (based on a mean SPF-IL score of 2.56 [SD = 0.45]; alpha (two-sided) = 0.05, beta = 0.20, ratio 1:1).

### Statistical analyses

Descriptive statistics are used to describe the study population at the two time points in the evaluation study (baseline and 12 month follow-up). Baseline variables are compared to detect differences between patients and professionals in the intervention group (FFF approach) and control group (care as usual). To assess baseline differences between the groups we use unpaired Student’s t-tests (continuous variables with normal distributions), Mann-Whitney U-tests (continuous variables with non-normal distributions) and Chi-square tests (categorical variables). Effect analyses are performed based on the intention-to-treat principle. Analyses of outcomes are performed by means of univariate, multivariate, and multilevel methods (to account for the nested structure of the data). To analyze the differences in outcomes between the intervention group and control group, we employ linear mixed model with random effects (multilevel analysis). To estimate the effects of the FFF approach after 12 months a difference in differences model will be used followed by a sensitivity analysis method specifically developed for difference in differences model based on more general methods of bounds developed by Rosenbaum [138]. Potential confounding and effect modification is accounted for when performing the analyses and, if necessary, adjustments for baseline differences are made. To handle missing data multiple imputation techniques will be used. Ultimate goals of these analyses are to test the assumptions of the theoretical framework with the instruments described in the study protocol. Finally, we will assess clinical relevance of improvements made in cognitions and behaviors among both patients and professionals. The software package IBM SPSS statistics version 23 is used for all statistical analyses.

### Timeline

**Fig. 2:**
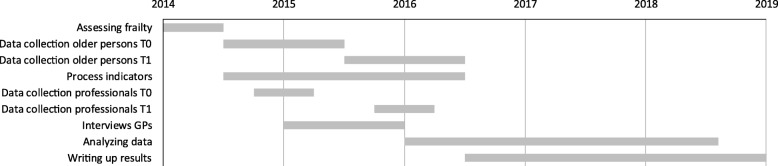
Timeline

Figure 2 shows a general timeline of the data collection among older adults and healthcare professionals, the analyses of the data and writing up the results of the evaluation study.

## Discussion

Integration of health services is increasingly advocated as a means to develop more effective models of care and improve patient outcomes [37]. Much research in the field of integrated care for community-dwelling older adults has been conducted, however, these innovative interventions have had mixed effects on patient outcomes and there is a need for in-depth evaluations. This underlines the importance of sound theory-based evaluations of integrated primary care approaches. Consequently, in efforts to evaluate the effects of innovative integrated care approaches, insight into the underlying mechanisms explaining the (lack of) effectiveness of these complex multicomponent interventions is crucial. The present paper describes the design of a theory-based evaluation of a proactive integrated primary care approach to improve well-being among frail community-living older adults.

### Strengths and limitations

A major strength of the study is the comprehensive and rigorous evaluation of the complex multicomponent integrated care approach FFF. We use a combination of quantitative and qualitative research methods and assess not only the effectiveness of the approach on frail older persons’ well-being, but also the cost-effectiveness and processes. Selected outcome measures are based on the theoretical model, which facilitates a sound theory-based evaluation. We ultimately may reveal crucial underlying mechanisms of this integrated care approach. Therefore, the theory-based evaluation study is expected to contribute to the existing evidence on improvements in quality of care and patient outcomes, and a better understanding of explanatory mechanisms underlying integrated primary care approaches.

The proposed evaluation study has potential limitations and challenges. First, the absence of randomization makes the design more susceptible to bias [139]. Especially selection bias is a major concern in non-randomized studies. Systematic differences between the groups result in incomparable groups which ultimately may lead to biased estimates of the intervention effect [140]. To reduce the impact of this bias on the outcome measures studied, we aim to control for important factors in the analysis of the data and by means of matching [141]. To ensure that the intervention and control groups are similar for key covariables, we use one-to-one matching to balance groups instead of matching on a higher level (at healthcare practice level). Moreover, when necessary we use case-mix adjustments to take into account important dissimilarities. However, it is stated by Deeks and colleagues [140] that the degree to which techniques can sufficiently adjust for differences between the groups is still unclear, which ultimately provides no guarantee for unbiased study results [140]. In addition, unknown and unmeasured factors can still influence the outcome [142]. Second, the design of the study makes it impossible to blind participating healthcare professionals and frail older patients. Knowledge of the status of the person (receiving either the FFF approach or care as usual) may have an influence on the responses and may affect compliance [143]. Nevertheless, the interviewers that conduct the interviews with frail older persons are kept unaware of the group the person is in (intervention or control GP practice), so that the interviewers collecting outcome data are not influenced by that knowledge. Blinding of the interviewers aids to reduce differential outcome measurements (information bias) [143]. Due to the nature of the evaluation study, however, it is possible that the patient inadvertently reveals his or her status during the interview (e.g. disclosing information that is specific to the FFF approach). Third, one of the core challenges of the evaluation study is the willingness of frail community-dwelling older patients to participate in the study, especially in the long-term. Recruitment of appropriate numbers of patients requires a sufficiently long period [144]. We aim to optimize participation in the evaluation study by means of home visits instead of interviews over the telephone, recruiting interviewers that live in the same region as the older adults, and sending letters to older patients on behalf of their own GP. Fourth, although control GP practices continue to provide usual care, GPs in the control group may start initiatives to improve care delivery for frail older patients. We collect data on various interventions that are implemented to improve care for older adults and we monitor and describe the activities performed by the GPs. In contrast to the intervention GP practices, control practices are not supported financially by the health insurers to implement elements of the FFF approach. Fifth, recall bias may potentially affect our study findings. Earlier research using a 12 months period of asking patients about their healthcare visits show both under-reporting and over-reporting effects [145]. Administrative data could be included to accurately capture resources for an economic evaluation (if filled in correctly). Sixth, while we included patients and professionals in our theoretical framework and study protocol we did not include informal caregivers. Given their important role in supporting community-dwelling frail older people they are expected to influence the well-being of older persons as well. Given the complexity of the theoretical framework as presented in this paper we decided to first unravel the underlying mechanisms in the relationship between quality of care, cognitions and behaviors of patients and professionals and older persons’ well-being. Future research should look at the role of informal caregivers and their cognitions and behaviors as well. It may be easier to improve outcomes if the patient’s partner is a good self-manager with a positive frame of mind compared to a partner who is depressed, has low self-efficacy and poor investment behavior.

## Abbreviations

ACIC-S: Assessment of Chronic Illness Care Short version
CCM: Chronic Care Model
COPD: Chronic Obstructive Pulmonary Disease
EQ-5D-3L: EuroQol (5 dimensions and 3 levels)
FFF: Finding and Follow-up of Frail older persons
GP(s): General practitioner(s)
ICER(s): Incremental cost-effectiveness ratio(s)
Katz ADL index: Katz Activities of Daily Living index
MMSE(-12): (12-item) Mini-Mental State Examination
PACIC-S: Patient Assessment of Chronic Illness Care Short version
QALY(s): Quality-Adjusted Life-Year(s)
SD: Standard deviation
SF-20: 20-item Short Form Health Survey
SFSPC-model: Model for reporting on Somatic, Functional, Social, Psychological, and Communicative indications
SMAS-S: Self-Management Ability Scale Short version
SPF-IL: Social Production Function Instrument for the Level of well-being
TFI: Tilburg Frailty Indicator
